# Isolation, Characterization, and Complete Genome Sequence of Escherichia Phage KIT06 Which Infects Nalidixic Acid-Resistant *Escherichia coli*

**DOI:** 10.3390/antibiotics13070581

**Published:** 2024-06-23

**Authors:** Nguyen Song Han, Mana Harada, Nguyen Huan Pham-Khanh, Kaeko Kamei

**Affiliations:** 1 Department of Functional Chemistry, Kyoto Institute of Technology, Kyoto 606-8585, Japan; nguyensonghan.kit@gmail.com (N.S.H.); h.m.5a.cookie@gmail.com (M.H.); 2 Department of Biology, College of Natural Sciences, Can Tho University, Can Tho City 900000, Vietnam; pknhuan@ctu.edu.vn

**Keywords:** *Escherichia* phage, phage virions, nalidixic acid-resistant *E. coli*, phage receptors, antibiotic-resistant *E. coli*

## Abstract

*Escherichia coli* (*E. coli*) is one of the most common sources of infection in humans and animals. The emergence of *E. coli* which acquires resistance to various antibiotics has made treatment difficult. Bacteriophages can be considered promising agents to expand the options for the treatment of antibiotic-resistant bacteria. This study describes the isolation and characterization of *Escherichia* phage KIT06, which can infect *E. coli* resistant to the quinolone antibiotic nalidixic acid. Phage virions possess an icosahedral head that is 93 ± 8 nm in diameter and a contractile tail (116 ± 12 nm × 13 ± 5 nm). The phage was found to be stable under various thermal and pH conditions. A one-step growth curve showed that the latent time of the phage was 20 min, with a burst size of 28 particles per infected cell. Phage KIT06 infected 7 of 12 *E. coli* strains. It inhibited the growth of the host bacterium and nalidixic acid-resistant *E. coli*. The lipopolysaccharide and outer membrane proteins of *E. coli*, *tsx* and *btuB*, are phage receptors. Phage KIT06 is a new species of the genus *Tequatrovirus* with a genome of 167,059 bp consisting of 264 open reading frames (ORFs) that encode gene products related to morphogenesis, replication, regulation, and host lysis. The lack of genes encoding integrase or excisionase indicated that this phage was lytic. Thus, KIT06 could potentially be used to treat antibiotic-resistant *E. coli* using phage therapy. However, further studies are essential to understand its use in combination with other antimicrobial agents and its safe use in such applications.

## 1. Introduction

Bacterial antimicrobial resistance (AMR) is a major threat to public health in the 21st century. An estimated 4.95 million deaths were associated with bacterial AMR worldwide in 2019 [1]. *E. coli* is ranked first among the six leading pathogens responsible for deaths [1]. *E. coli* is resistant to various antibiotics, making infections caused by this bacterium difficult to treat in humans and in veterinary medicine worldwide [2]. Alternative therapeutic methods are needed to expand treatment options for antibiotic-resistant bacteria. Bacteriophages are promising therapeutic agents.

Bacteriophages (also known as phages) are viruses that specifically lyse bacteria. They are ubiquitous, with a prevalence of 10^31^ on Earth [3]. They were discovered more than a century ago and have been studied for their potential as antimicrobial agents. However, interest in phage applications declined with the discovery of penicillin in 1928 and the advent of antibiotics as therapeutic agents in the 1940s [4]. In recent years, the rise of antibiotic-resistant bacteria has renewed interest in the use of phages as alternatives to antibiotics to control these pathogens [5].

Phages initiate infection by attaching to the surface of bacterial cells via specific receptors. Subsequently, genetic material is injected into the host cell. Once inside, they either integrate their genome into the bacterial chromosome and replicate with the bacterium (lysogenic infection) or utilize the bacterial replication machinery to produce new generations of phages (lytic infection) [6].

Phages possess several advantages over antibiotics as antimicrobials, including a limited occurrence of secondary infections during their application, specificity for the bacteria they infect, and an ability to infect antibiotic-resistant bacteria [7]. T-even phages (T2, T4, and T6) possess a strictly lytic lifestyle (virulent phages) that degrades host chromosomes during host cell infection. Furthermore, they typically possess a broad host range for pathogenic bacteria [8]. Based on classification by the International Committee Taxonomy on Viruses, these phages are *Tequatroviruses* belonging to the *Straboviridae* family.

Nalidixic acid is a quinolone antibiotic that has been synthesized and suggested for treating urinary tract infections. Nalidixic acid has bactericidal and bacteriostatic effects depending on the concentration and sensitivity of the microorganisms. This substance affects gram-negative microorganisms, including *E. coli* [9]. Alterations in genes that encode DNA gyrase (*gyrA* and *gyrB*) and topoisomerase IV (*parC* and *parE*) are the most frequent mechanisms of quinolone resistance in *E. coli*. Alterations in *gyrA* and its homologous region in *parC* are involved in quinolone resistance. In contrast, mutations in *gyrB* and *parE* are minor and rarely contribute to quinolone resistance [10,11,12,13]. This study found a large variety of amino acid changes in *gyrA* and *parC* proteins in nalidixic acid-resistant *E. coli* strains isolated from food products, humans, and animals [11].

A few studies on bacteriophages against nalidixic-resistant *E. coli* have been reported. Phage vB_Ec_ZCEC14 could infect clinical *E. coli* strains that were resistant to multiple antibiotics, including nalidixic acid [14]. Another group reported that ten phages could infect *E. coli* strains, including those resistant to nalidixic acid, in both in vitro experiments and a mouse model [15].

In this study, we screened *Escherichia coli* phages using several *E. coli* strains as hosts to identify new phages. We successfully isolated phage KIT06, which formed clear and round plaques on *E. coli* NBRC 3972. We analyzed host specificity and found that phage KIT06 lysed nalidixic acid-resistant *E. coli*. Thus, this study aimed to identify and characterize phage KIT06. We present the latent period, burst size, host range, binding receptors on the bacterial membrane, pH and thermal stability, bacteriolytic activity, and genomic and phylogenetic characteristics of *Escherichia* phage KIT06. Understanding these properties will help determine the suitability of phage KIT06 as a potential agent for combating harmful bacteria.

## 2. Results

### 2.1. Morphology of Escherichia Phage KIT06

**Figure 1:**
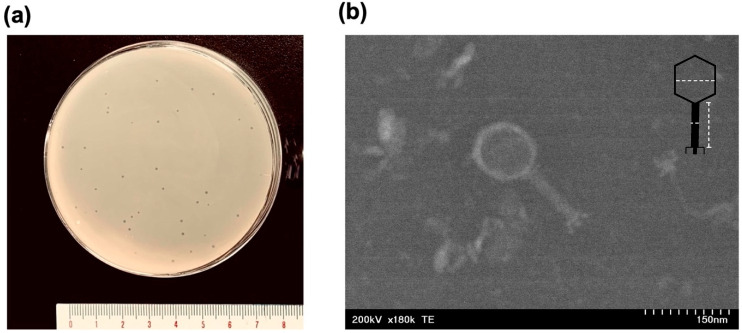
Plaques formed by *Escherichia* phage KIT06 on the lawn of *E. coli* NBRC 3972 (**a**). Electron micrograph image of phage, scale 150 nm (**b**), the dashed lines indicated measurement of the diameter of the head and length and width of the tail.

Bacteriophage KIT06, isolated from duck intestine, displayed distinct round plaques measuring approximately 1 mm in diameter on an overlay seeded with *E. coli* NBRC 3972 (Figure 1a). The morphology of phage KIT06 was observed using scanning transmission electron microscopy (STEM). The virion structure of KIT06 exhibited an isometric head with a diameter of 93 ± 8 nm and a contractile tail measuring approximately 116 ± 12 nm in length and 13 ± 5 nm in width (Figure 1b). KIT06 shares the characteristics of a myovirus with a contractile tail compared to previous studies on phages KIT03 and PS5 [16,17]. However, these phages have only been isolated from diverse sources and geological locations.

### 2.2. Host Specificity

The host specificity of phage KIT06 was investigated by testing its infectivity against 12 *E. coli* strains, including antibiotic-resistant variants (Table 1). Bacteriophage KIT06 infected seven *E. coli* strains, including the nalidixic acid-resistant *E. coli* ATCC 700609 (58% of the 12 tested strains). The efficiency of plating (EOP) of the phages was determined using a plaque assay, with the highest EOP value observed in phages infected with *E. coli* NBRC 12062 (Supplementary Table S1).

### 2.3. One-Step Growth Curve Experiment

**Figure 2:**
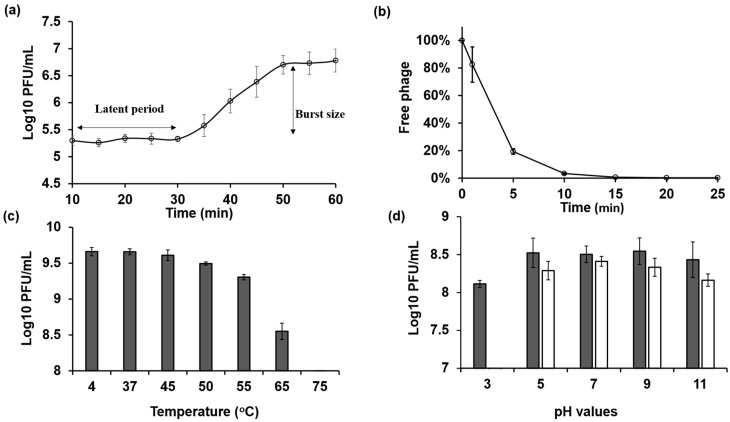
(**a**) One-step growth curve of KIT06 phage on host *E. coli* NBRC 3972 was drawn from 10 min after 5 min adsorption of phage to bacteria followed by centrifugation to remove free phage. (**b**) The adsorption rate of phage KIT06 to host bacteria NBRC 3972 was measured by counting unadsorbed phage particles. (**c**,**d**) The stability of phage KIT06 was evaluated as its infection ability on *E. coli* NBRC 3972 after exposure to various temperatures (**c**) for 1 h and various pH values (**d**) for 1 h (dark gray) and 24 h (white). Data are represented as mean ± SD (*n* = 3).

The one-step growth curve experiment was carried out by infecting the host bacteria NBRC 3972 with phage KIT06 at a multiplicity of infection (MOI) of 0.01, a latent period of 20 min, and a burst size of 28 particles per infected cell (Figure 2a).

### 2.4. Phage Adsorption

After 1 min of adsorption of Escherichia phage KIT06 to the host bacteria NBRC 3972, we counted the free phages in the filtrate. The results indicate that 83% of the initial phage particles were not adsorbed to the host bacteria. The amount of unadsorbed phages was significantly reduced to 19% after 5 min of adsorption. The number of free phages gradually decreased to 3% and 1% at 10 and 15 min, respectively. No free phages were observed at 20 or 25 min after infection, indicating that the phages were completely adsorbed into the bacterial cells by 20 min after infection (Figure 2b).

### 2.5. Stability of Escherichia Phage KIT06

The highest thermal stabilities of phage KIT06 were observed at 4 °C (control) and 37 °C (Figure 2c). The phage activity slightly decreased at 45 °C but maintained a high titer. The phage lost its activity completely after 1 h of incubation at 75 °C. The survivability of the phage was also assessed by incubation for 1 or 24 h under different pH conditions ranging from acidic to alkaline (Figure 2d). The phage was stable at pH 5–11 after a 1-h incubation. However, there was an approximately 0.5 log plaque-forming units per mL (PFU/mL) reduction in phage infection ability at pH 3 compared to other pH values. The phage exhibited the highest stability at pH 7 following a 24 h incubation, whereas slightly lower activity was observed at pH 5 and 9. Stability gradually decreased at pH 11. Notably, the phage completely lost stability at pH 3. These findings suggest that KIT06 is stable under various pH and temperature conditions.

### 2.6. Bacteriolytic Activity of the Phage

**Figure 3:**
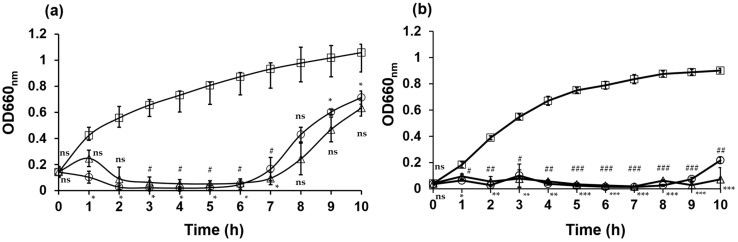
Bacteriolytic activities of phage KIT06 on host bacteria *E. coli* NBRC 3972 (**a**) and nalidixic acid-resistant *E. coli* ATCC 700609 (**b**). Bacteria were cultured with phage KIT06 at MOI 0.1 (triangle) and 1 (circle), or without phage as a control (square), and bacterial growth was monitored by measuring OD_660 nm_. Data are represented as mean ± SD (*n* = 3) and the statistical significance between bacterial growth with and without phage KIT06 was analyzed by two-way ANOVA and Dunnett test. *p*-value, *^, #^, *p* < 0.05; **^, ##^, *p* < 0.01; ***^, ###^, *p* < 0.001; ns, non-significant (*p* > 0.05). *, at MOI 0.1; #, at MOI 1.

The lytic activities of the bacteriophages against *E. coli* NBRC 3972 and nalidixic acid-resistant *E. coli* ATCC 700609 at MOIs of 1 and 0.1 were determined using previously described methods with slight modifications [18]. Phage KIT06 inhibited bacterial growth at both MOIs (Figure 3). In the case of *E. coli* NBRC 3972 as a host bacterium, the phage suppressed bacterial growth from 1 to 7 h post-infection. The optical density (OD) at 660 nm remained below 0.1 during this period. However, bacterial growth in both MOI groups started to recover from approximately 7 h post-infection, reaching an OD_660nm_ of 0.6 at 10-h post-infection (Figure 3a).

KIT06 suppressed the growth of nalidixic acid-resistant *E. coli* 1 h after incubation and maintained this activity until 9 h after incubation. The optical density remained below 0.1 for both MOIs during this period. However, bacterial growth resumed at 10 h post-infection, reaching an OD_660nm_ 0.2 of MOI 1 (Figure 3b). Our data suggest that the phage inhibits the growth of *E. coli* NBRC 3972 at MOI 1 more quickly at the beginning of infection compared to at MOI 0.1. However, the regrowth of bacteria was observed at approximately 6 h post-infection at an MOI of 1, which was earlier compared to the regrowth at an MOI of 0.1. Overall, these findings demonstrate the efficacy of phage KIT06 in inhibiting bacterial growth within several hours of infection and that growth inhibition was more effective for nalidixic acid-resistant *E. coli*. The lytic activities of KIT06 at the two MOIs were similar to those of phage VB_EcoS-Golestan against *E. coli* 333, which retained its lytic activity until 8 h of incubation [19].

### 2.7. Bacteriophage Receptors

**Figure 4:**
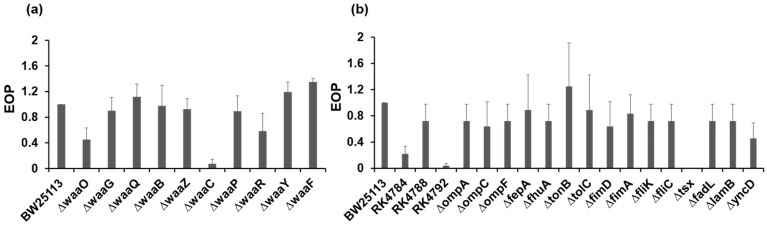
Infection of KIT06 to LPS mutants (**a**) and mutants of outer membrane proteins or flagellar mutants (**b**). Infection was shown with EOP. Data are represented as mean ± SD (*n* = 3).

The host receptors to which the phage bound for the infection were investigated using the Keio collection, which consists of single-gene deletion mutants related to lipopolysaccharide (LPS) synthesis, outer membrane proteins, or flagellar proteins of *E. coli* BW25113 strain (Supplementary Tables S2 and S3), and *btuB* deletion mutants (Supplementary Table S4). Plaque assays were performed to evaluate the infection abilities of phage to the mutants, and EOP was calculated using the reference strain, *E. coli* ME9062 (BW25113 strain). Among the core oligosaccharide (OS) mutants of LPS, the KIT06 phage showed the lowest infection rate with the Δ*waaC* mutant, which exhibits a truncation in the inner core OS and deficiencies in heptose (Hep) I, Hep II, and Hep III (Figure 4a, Supplementary Figure S1 and Supplementary Table S2). The Δ*waaO* and Δ*waaR* mutants, which lack Glc II and Glc III in the outer core OS, respectively exhibited lower susceptibility to the KIT06 phage. Additionally, the KIT06 phage showed no infection to deletion mutant of *tsx,* which encodes the nucleoside-specific channel-forming protein *tsx*, and weak infection with the *E. coli* RK4792 strain, which is defective in the vitamin B12 transporter (Figure 4b, Supplementary Tables S3 and S4).

### 2.8. Whole Genome Sequencing

**Figure 5:**
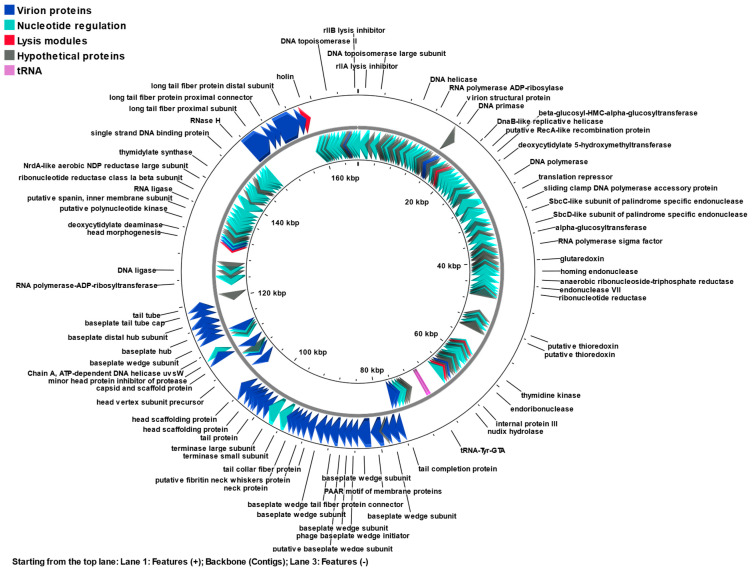
Genome map of phage KIT06 containing 264 identified ORFs. Different colors represent functional categories: virion proteins (blue), nucleotide regulation proteins (green), lysis modules (red), hypothetical proteins (gray), and tRNAs (magenta). The map was generated by Proksee.

The KIT06 genome comprised 167,059 base pairs using a de novo genome assembler. The G + C percentage of the genome was 35.6%. Based on the whole-genome sequence, the bacteriophage KIT06 was classified as a myovirus belonging to the class *Caudoviricetes*. Two hundred and sixty-four predicted open reading frames (ORFs) were identified in the genome of this phage (Supplementary Tables S5 and S6). The whole genome map and functional proteins of KIT06 showed various gene products, such as virion proteins, nucleotide regulation proteins, lysis proteins, and hypothetical proteins (Figure 5). These proteins are predicted to play important roles in virion morphogenesis, DNA replication, transcription, and host lysis. Ten tRNAs with the anticodons Gln, Leu, Gly, Pro, Ser, Thr, Met, Tyr, Asn, and Arg were included. No integrated genes, virulence genes, or antibiotic-resistance genes were detected in the genome of phage KIT06. In total, 138 ORFs (52.3%) were defined as functional proteins based on their homology with known sequences in various databases. In addition, 126 ORFs were predicted to encode hypothetical proteins.

#### 2.8.1. Nucleotide Regulation

DNA replication, recombination, and repair by phage KIT06 may follow the replication mechanism of phage T4 [20], which is regulated by a single-stranded DNA binding protein (ORF233), sliding clamp DNA polymerase (ORFs 51, 53), DNA helicase loader (ORF234), DNA helicase (ORF181), and UvsY (ORF104). Thymidylate synthase is encoded by ORF226, which catalyzes the synthesis of DNA precursors. Furthermore, ORFs 84, 92, and 94 encode thioredoxins involved in phage-induced ribonucleotide reduction. Recombination endonuclease VII in the KIT06 genome cleaves cruciform and Y-structure, repairs mismatches, and assists in DNA packaging by removing replicative branches. Notably, phage KIT06 possesses an rIIA lysis inhibitor encoded by ORF1, which is crucial for viral replication within clusters carrying different enzymes that synthesize deoxyribonucleotides and are associated with the host DNA and membrane.

On the genome of phage KIT06, there is one homing endonuclease encoded by ORF77 and three predicted endonucleases (ORFs 222, 262, and 263) which are specific to the thymidylate synthase gene splice junction and are involved in intron homing. Proteins involved in the transcription process of phage KIT06 including RNA polymerase (ORF64) may play a role in DNA packaging by interacting with the terminase subunit. The anti-sigma factor (ORF244) plays an important role in the pre-replication period of phages.

#### 2.8.2. Virion Proteins

At least 48 ORFs were predicted to be virion proteins, which construct the head, neck, tail sheath, and tail fibers as the basic structures of the myovirus. The major head and scaffolding proteins were predicted to function as ORFs 170, 171, 172, 173, and 179. ORF30 and ORF260 encode the small outer capsid and outer membrane proteins, respectively. ORFs 29, 147, 175, 180, and 208 were used to construct the phage head. Tail proteins involving the tail sheath (ORFs 163 and 166), tail fiber proteins (ORFs 159, 210, 238, 239, and 241), and other tail proteins encoded by the other four ORFs were identified. Several ORFs, such as 160, 161, and 162, encode the fibritin neck whisker and neck proteins, respectively. Putative baseplate hub and base plate wedge subunit proteins are encoded by ORFs 148, 149, 152,153, 154, 155, 156, 157, 158, 186, 187, 188, 189, 190, and 191. Furthermore, the three components, small terminase, large terminase, and portal protein, are encoded by ORFs 164, 165, and 168, respectively, in the T4 phage genome-packaging machine.

#### 2.8.3. Lysis Modules

The phage possesses endolysin and holin encoded by ORFs 124 and 243, respectively, which are the most common lytic enzymes that release progeny virions at the final stages of the lytic cycle, leading to host lysis. Holin oligomerizes, resulting in the formation of pores in the cytoplasmic membrane that allow the endolysin to be released into the periplasm. Endolysins are peptidoglycan hydrolases that digest peptidoglycans in bacterial cell walls. Holin is regulated by a lysis inhibition regulator encoded by ORF107. The genomic DNA from the superinfecting phage binds to holin-anti-holin, probably signaling lysis inhibition and blocking holin multimerization [21,22,23].

### 2.9. Phylogenetic and Genome Comparative Analysis of KIT06

**Figure 6:**
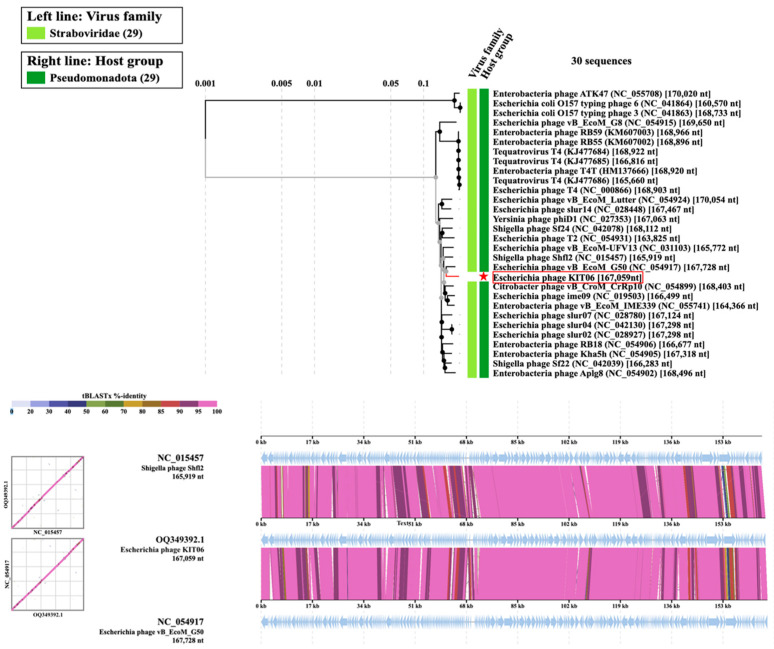
Phylogenic tree of the entire genome. *Escherichia* phage KIT06 was marked with a red star (**upper**). Comparing the genomes of *Escherichia* phage KIT06, *Shigella* phage Shfl2, and *Escherichia* phage vB_EcoM_G50 (**bottom**).

A phylogenetic tree of the complete KIT06 genome was constructed using the viral proteomic tree server (Viptree). Among the related phages, KIT06 showed the closest relationship to two phages belonging to *Tequatrovirus Shigella* phage JK23 (MK962752.1), with 92% query coverage and 96.28% identity, and *Escherichia* phage vB_EcoM_G50 (MK327942.1), with 95% query coverage and 97.95% identity. Bacteriophage KIT06 is a new species of this genus, owing to the <95% homology of its entire genome to that of other species [24]. Other phages, including *Shigella* phage Shfl2, *Escherichia* phage vB_EcoM_UFV13, *Escherichia* phage T2, *Citrobacter* phage vB_CroM_CrRp10, and *Escherichia* phage ime09, also belong to the *Tequatrovirus* genus (Figure 6).

## 3. Discussion

This study describes the isolation and properties of *Escherichia* phage KIT06 from duck intestines. Previous studies have reported the isolation of bacteriophages from different environmental sources, such as soil, sewage, chicken products, feces, and animal offal. For instance, bacteriophages vB_PmiS-TH and VB_EcoS_Golestan were isolated from sewage sources in Iran; and bacteriophages SP5 and KIT03 were isolated from chicken products and soil, which can simultaneously infect *Salmonella* and *E. coli* O157:H7 [16,17,19,25]. Various bacteriophages infecting *E. coli* and six lytic phages for controlling pathogenic *E. coli* have been isolated from chicken and beef offal [26], whereas a polyvalent phage (PhiLLS) has been isolated from pond water [27].

KIT06 can infect various *E. coli* strains such as strains B, Crooks, BL21, BW25113, and nalidixic-resistant *E. coli*. A strength and significant challenge of phage therapy is the specific host range of phages that can infect a single bacterial species or even a bacterial strain [28]. It is a strength since it allows the selection of phages that target selected problematic bacteria; however, a combination of phages with different host ranges may need to be prepared to broaden the lytic spectrum for phage therapy to target a panel of relative bacteria [29]. Nevertheless, this mixture may lead to recombination and antagonism among phage members and increase the cost of phage isolation, characterization, and production [30,31,32]. Phage KIT06 may need to be used with other phages for phage therapy applications to target other related strains of *E. coli*. *E. coli* NBRC 3972, used to isolate phage KIT06, had no toxic genes, such as *stx*1 (Shiga toxin 1) or *stx2* (Shiga toxin 2), in the bacterial genome. Phage KIT06 contained neither toxic nor integrated genes in its genome. However, further studies are needed to confirm the safety of these applications, including phage therapy.

EOP values varied among the susceptible strains (Supplementary Table S1). Modification of surface molecules that function as phage receptors can significantly affect the adsorption of phages onto bacteria. Consequently, the notable variations in EOP were due to differences in the receptors of the strains [33,34]. Our results are probably similar to those of a study on *Pseudomonas* phage BrSP1, with differences in EOP values of up to nearly a hundred-fold among the bacteria strains [35].

Analysis of the morphology of the phage KIT06 using STEM revealed that it is a myovirus, similar in structure to phages KIT03 and PS5 studied previously, characterized by an icosahedral head and contractile tail. However, these phages were isolated from diverse sources and environments [16,17].

The latent period of KIT06 was 20 min with a burst size of 28 PFU per cell. The latent period of this phage is similar to that of other phages but has a smaller burst size. Phage PS5 possessed latent times of 20, 25, and 25 min, and estimated burst sizes of 148, 98, and 204 PFU per cell for infecting *E. coli* O157:H7, *Salmonella enteritidis*, and *Salmonella typhimurium*, respectively [17]. Phage VB_EcoS-Golestan had a latent period of 40 min, with a burst size of approximately 100 PFU per cell [19]. In general, a phage with a short latent period and high burst size can overcome phage competition and may be more effective in phage therapy [27,36,37]. Phage KIT06 was stable at various temperatures and pH values, indicating that it could be used for phage therapy. The adsorption rate of phage KIT06 on the host bacterial strain reached 99% at 15 min post-infection. This result was similar to that of phage VB_EcoS-Golestan, which showed an adsorption rate of 99.5% 15 min after adsorption. On the other hand, *Escherichia* virus myPSH2311 showed complete adsorption 30 min after infection [19,38].

*Escherichia* phage KIT06 inhibited the growth of *E. coli* NBRC 3972 and nalidixic acid-resistant *E. coli* at an MOI of 0.1 and MOI 1, as shown in Figure 3. However, *E. coli* NBRC 3972 and nalidixic acid-resistant *E. coli* started to grow again at 7 and 10 h post-infection, respectively. These results are similar to those of a previous study on phages SP5 and PP01, which infect *E. coli* 0157:H7 (ATCC 43888), and an increase in turbidity was observed at approximately 8 h, likely due to the emergence of phage-resistant bacteria [39]. Host bacteria treated with phage KIT06 at MOI 1 regrew earlier than those treated at MOI 0.1, suggesting that a lower concentration of phage suppresses the generation of phage-resistant bacteria. We do not know the reason for this, but this result is similar to that of phage KIT04, which infects *Vibrio parahaemolyticus*. The increase in bacterial numbers started again at approximately 9 and 11 h after treatment with phage KIT04 at MOI 1 and MOI 0.01, respectively [40]. Phage vB_EcoStr-FJ63A, which infects colistin-resistant *E. coli*, was examined for bactericidal activity against the host bacterium, *E. coli* 63, at MOIs ranging from 0.01 to 100. The host bacteria treated with higher concentrations of phage vB_EcoStr-FJ63A started to regrow earlier and grew faster than bacteria treated with lower concentrations of the phage [41].

The 167,059 bp genome of KIT06 contained 264 ORFs. None of the gene products of these ORFs have been implicated in lysogenic or antibiotic resistance. The phage was homologous to other phages belonging to the genus *Tequatrovirus,* with its genome possessing a size and composition similar to those of ORFs. Compared with other phages in the *Tequatrovirus* genus, the phage KIT06 genome is from hundreds to thousands of base pairs different from the 167,728 bp genome with 267 coding sequences of *Escherichia* phage vB_EcoM-G50, and the 168,349 bp genome with 272 coding sequences of *Shigella* phage JK23 [42,43].

We also clarified the receptor molecules of phage KIT06 using various mutants deficient in LPS, outer membrane proteins, and flagellar proteins. A bacteriophage first recognizes and binds to one or more receptors on the surface of susceptible bacterial cells [44]. In gram-negative bacteria, phages bind to specific proteins and/or lipopolysaccharide (LPS) moieties present on the outer cell membrane. In the analyses of receptor molecules using various mutant bacteria, phage KIT06 did not infect the *waaC* deficient mutant and showed less infection to Δ*waaO* and Δ*waaR* mutants. These results suggest that KIT06 binds to Hep I in the inner core OS of LPS, together with weaker binding to Glc I and Glc II in the outer core OS at the initial infection step (Supplementary Figure S1). In contrast, Δ*waaF,* Δ*waaY,* and Δ*waaQ* mutants seem to show a larger susceptibility to phage KIT06. This suggests that Hep II, the phosphate group linked to Hep II and Hep III, disturbed the adsorption of phages on *E. coli* LPS. Similar to our data, in a previous study, the identification of phage receptors using *Yersinia pestis* mutant strains showed higher EOP values than those of the parent strain [33]. Furthermore, KIT06 utilizes *tsx* and *btuB* membrane proteins as secondary receptors. The attachment mechanism of bacteriophage KIT06 is likely similar to that of phage T6, both belonging to the genus *Tequatrovirus*, which attaches to the *tsx* nucleoside channel of *E. coli* [45].

The emergence of antibiotic resistance makes phage therapy an alternative approach to overcoming multidrug-resistant bacteria. Although the phage was screened using *E. coli* NBRC 3972 as the host, we discovered that phage KIT06 could lyse nalidixic acid-resistant *E. coli*. To the best of our knowledge, phages infecting nalidixic acid-resistant *E. coli* have been reported in only a few studies [14,15]. The receptor molecules of the ten phages closely related to KIT06 in the genome sequence shown in Figure 6 have not been reported. Thus, this study provides novel information regarding the phages that lyse nalidixic acid-resistant *E. coli*. Phage KIT06 may be useful for the treatment of multiple drug-resistant *E. coli* infections in combination with other phages with different host specificities. Although KIT06 contains neither toxic nor integrase genes in its genome, further studies are needed to confirm the safety of its applications, including phage therapy.

## 4. Materials and Methods

### 4.1. Bacteria

The *E. coli* strains listed in Table 1 were obtained from the NBRC (Biological Resource Center, National Institute of Technology and Evaluation, Tokyo, Japan) or the ATCC (American Type Culture Collection, Manassas, VA, USA). *E. coli* NBRC 3972, used to isolate phage KIT06, had no toxic genes, such as *stx*1 (Shiga toxin 1) or *stx2* (Shiga toxin 2), in the bacterial genome.

Lipopolysaccharide (LPS) mutants of *E. coli* (Keio collection) are listed in Supplementary Table S2, and mutants of membrane or flagellar proteins listed in Supplementary Tables S3 and S4 were obtained from the National Bioresource Project (NBRP, Shizuoka, Japan). Because the *E. coli* BW25113 strain is the parent strain for generating mutants in the NBRP, *E. coli* ME9062 (strain BW25113) was used in this study as a control strain to analyze receptor molecules. All *E. coli* strains were cultured in Luria–Bertani (LB) broth.

### 4.2. Sample Collection and Phage Isolation

The *E. coli* strain Crooks (NBRC 3972) was used as a host to isolate bacteriophages. Duck intestines were purchased from a local market in Can Tho City, Vietnam (Lat. 10.040188 N, long. 105.775312 E). Bacteriophages were isolated by precipitation [18] Briefly, ten grams of duck intestine was suspended in 25 mL of SM buffer (200 mM NaCl_2_, 10 mM MgSO_4_ and 50 mM Tris-HCl, pH 7.5), the supernatant was collected by centrifugation (6000× *g*, 4 °C, 10 min), added to final concentration of polyethylene glycol 6000 (15% *w*/*v*, FUJIFILM Wako Pure Chemical Corp., Osaka, Japan) and NaCl (2.9% *w*/*v*), and kept at 4 °C overnight. The precipitate was obtained by centrifugation and resuspended in SM buffer to obtain a crude phage solution. 

The crude phage was dropped onto the bacterial lawn which had been mixed with *E. coli* NBRC 3972 (10^8^ CFU/mL), and incubated at 37 °C overnight. Subsequently, the phage was collected from a clear zone formed on the agar. After mixing with soft agar, the phage was overlaid on the host bacteria, and a single plaque was picked at least three times to ensure purity [46]. Thus, *Escherichia* phage KIT06 was obtained.

### 4.3. Morphology Analysis

A high-titer phage suspension was spotted onto an elastic carbon support film on a copper grid (Okenshoji Co., Ltd., Tokyo, Japan). The bacteriophages were allowed to adsorb for 1 min and then stained with 1% (*w*/*v*) sodium phosphotungstate (pH 7.0) (Sigma-Aldrich, Burlington, MA, USA). The grids were dried overnight in a desiccator. The bacteriophages were observed using STEM (Scanning Transmission Electron Microscope HD-2700, Hitachi High-Technologies Corp, Tokyo, Japan) in TEM mode at 200 kV [47].

### 4.4. Host Specificity

The Escherichia phage KIT06 was dropped onto 12 *E. coli* strains, including antibiotic-resistant *E. coli*, and plaque formation was observed to determine host specificity. The EOP was also measured using a plaque assay [46]. This experiment was performed in triplicate.

### 4.5. One-Step Growth Curves

A one-step growth curve experiment was performed with some modifications to determine the latent period and phage burst size [48]. *E. coli* NBRC 3972 was cultured to the exponential phase. Subsequently, the phages were added at an MOI of 0.01 and allowed to absorb for 5 min at room temperature. The mixture was then centrifuged, and the pellet was resuspended in 10 mL LB broth and cultured at 37 °C, 150 rpm. The culture (100 µL) was taken at 5 min intervals until 60 min, mixed with fresh bacteria suspension and soft agar (0.5%), and overlaid on LB agar for the double layer agar–plaque assay method [46]. This experiment was performed in triplicate.

### 4.6. Phage Adsorption

Phage adsorption was conducted according to a previous report with slight modifications [49]. Briefly, the host bacterium *E. coli* NBRC 3972 was cultured in the exponential phase (approximately 10^8^ CFU/mL). The phage KIT06 was added to reach the MOI at 0.01 and shaken at 37 °C. The aliquots were taken at 0, 1, 5, 10, 15, 20, and 25 min after infection, and immediately filtered through 0.2 µm filters (ADVANTEC, Tokyo, Japan). Phages in the filtrates at different time points were counted using the double-layer agar–plaque assay method and expressed as a percentage of the initial phage count. The experiment was repeated three times.

### 4.7. Phage Stability

Phage KIT06 was incubated at 4, 37, 45, 50, 55, 65, and 75 °C for 1 h to determine its thermal stability. pH stability assays were performed by mixing the phage suspensions with a buffer containing 150 mM KCl, 10 mM Na_3_-citrate, and 10 mM boric acid, which was adjusted to pH 3, 5, 7, 9, or 11 using NaOH or HCl, followed by incubation for 1 and 24 h [50]. The phage titer was determined using the double-layer agar plaque assay method [46]. The experiment was performed in triplicate.

### 4.8. Bacteriolytic Characteristics of Phages

The bacteriolytic activity of the phages at different MOIs was determined using a previously described method with modifications [18]. An in vitro challenge test of the phages with two separate bacterial strains (*E. coli* NBRC 3972 and nalidixic acid-resistant *E. coli* ATCC 700609) was conducted. An overnight culture of each bacterium was inoculated in broth medium and further cultured at 37 °C under shaking. Bacteriophages with an MOI of 0.1 or 1 were added after one hour of culture. Bacterial growth was determined by measuring the optical density at 660 nm (OD_660_) every 1 h. The measurements were taken in triplicate.

### 4.9. Bacteriophage Receptors

The efficiency of plating was examined using bacterial mutant strains (Supplementary Tables S2–S4). Briefly, a ten-fold serial dilution of phage KIT06 was mixed with a mid-log phase of tested strains. The EOP was measured by plaque assay, and the results were normalized using *E. coli* K-12 BW25113 as the reference value of 1. The experiment was repeated three times.

### 4.10. DNA Genome Extraction

The genomic DNA of the phage was extracted according to a previous study, with slight modifications [51]. *E. coli* strain NBRC 3972 was infected with phage KIT06 and cultured at 37 °C for 7 h. After removing the bacterial cells and debris by centrifugation (8000× *g*, 4 °C, 10 min), the culture medium was filtered with a 0.2 µm membrane (ADVANTEC, Tokyo, Japan) and a solution containing 10% polyethylene glycol 6000 and 1 N NaCl was added at half the volume. The phages were collected as pellets by centrifugation and resuspended in 500 μL of 5 mM MgSO_4_. The suspension was treated with DNase I and RNase A (Sigma-Aldrich, Burlington, MA, USA) at 37°C for one hour, followed by the addition of Proteinase K (20 μg, Macherey-Nagel, Germany) and 0.5% sodium dodecyl sulfate (SDS)—20 mM ethylenediaminetetraacetic acid (EDTA), pH 8.0. The mixture was incubated further for 1 h at 60 °C then cooled to room temperature. An equal volume of phenol-chloroform-isoamyl alcohol (25:24:1) was added and the mixture was inverted several times. The mixture was centrifuged to remove the denatured protein. The phenol-chloroform-isoamyl alcohol step was repeated. A sodium acetate solution was added to achieve a final concentration of 0.3 M (pH 7.5). Subsequently, 2.5 volumes of ethanol were incorporated, and the resulting mixture was maintained at −20 °C overnight. After centrifugation, the precipitate was collected, washed with 70% ethanol, and dissolved in Tris-EDTA buffer to obtain the phage DNA.

### 4.11. Whole-Genome Sequence

Bacteriophage DNA was sequenced using an Illumina Miseq Paired-end (301 + 301) (Hokkaido System Science Co., Ltd., Hokkaido, Japan), and the raw data sequence was uploaded to the database with accession number PRJNA1101219. The output was assembled using de novo genome assembler Spades (version 3.11) with an average coverage of 153. Open reading frames (ORFs) were determined using the RASTtk web browser (https://rast.nmpdr.org/, accessed on 1 December 2022). The functions of the ORFs were annotated using the protein basic local alignment search tool (BLASTP) and the HHPred web server (https://toolkit.tuebingen.mpg.de, accessed on 23 December 2022). The tRNAs were determined using ARAGORN (v1.2.41), accessed on 10 May 2023 [52]. Abricate version 1.0.1 (https://github.com/tseemann/abricate, accessed on 16 May 2023) was used to detect the virulent genes of phage with seven databases, NCBI, CARD, Ecoli_VF, Resfinder, VFDB, MEGARES, and ARG-ANNOT [53,54,55,56,57]. A genome map was generated using Proksee (https://proksee.ca/, accessed on 1 May 2024). Phage genome alignments and a phylogenetic tree were generated using the Viptree web server https://www.genome.jp/viptree/, accessed on 1 February 2024 [58]. The phage genome was uploaded to GenBank with the accessory number OQ349392.1.

## Figures and Tables

**Table 1:** Host specificity of phage KIT06 on various strains of *E. coli*. The experiment was conducted in triplicate by the spot test. Results were recorded as –, no infection; +, infection. *, host strain used for the isolation of KIT06.

*E. coli*	Strain, Properties	Infection
NBRC 3972 *	Strain: Crooks	+
NBRC 12062	Strain: Ba15	+
NBRC 13168	Strain: B	+
NBRC 14129	Strain: W3110 lysogenized by phage 434	+
ATCC 43888	Serotype: O157:H7	–
ATCC 19110	Strain: CDC [NCTC9014], serotype: O14:K7(L):NM	–
ATCC 23506	Strain: NCDC Bi 8337-41, serotype: O10:K5(L):H4	–
BL21	Strain: BL21 derived from strain B	+
ME9062	Strain: BW25113 (substrain K12)	+
ATCC 700609	Strain: CN13, resistant to nalidixic acid	+
ATCC 700891	Strain: HS(pFamp)R, resistant to ampicillin and streptomycin	–
BAA-2469	Strain: 1001728, resistant to ertapenem and imipenem	–

## Data Availability

The genome sequences from this study are available in the GenBank database (accession number: OQ349392.1, https://www.ncbi.nlm.nih.gov/nuccore/OQ349392.1, accessed on 1 May 2024). Raw sequencing data were obtained from the SRA database (accession number: PRJNA1101219, https://www.ncbi.nlm.nih.gov/bioproject/PRJNA1101219/, accessed on 1 May 2024). All data supporting the conclusions of this study are included in the manuscript and Supplementary Materials.

## Supplementary Materials

The following supporting information can be downloaded at: https://www.mdpi.com/article/10.3390/antibiotics13070581/s1, Figure S1: Structure of the lipopolysaccharide (LPS) core oligosaccharide (OS) of *E. coli* K-12, glycosyltransferases genes (red), and core modification-related genes (blue) responsible for core OS synthesis; Table S1: Efficiency of plating (EOP) of *Escherichia* phage KIT06 on various *E. coli* strains; Table S2: Keio collection of single-gene knockout strains used in this study related to lipopolysaccharide (LPS) synthesis in the *E. coli* K12 BW25113 strain background. The LPS structure is taken from the website of the National BioResource Project (https://shigen.nig.ac.jp/); Table S3: Keio collection of single-gene knockout strains related to outer membrane proteins or flagella proteins in the *E. coli* K12 BW25113 strain background used in this study; Table S4: ME collection gene mutants used in this study, *btuB* protein mutants—vitamin B12 transporter; Table S5: Phage KIT06 genome annotation, the function of 264 ORFs, and 10 tRNAs; Table S6: Classification of putative ORFs in *Escherichia* phage KIT06 genome by predicted function. tRNAs are not included.
