# Supplementary material for: Isolation, Characterization, and Complete Genome Sequence of Escherichia Phage KIT06 Which Infects Nalidixic Acid-Resistant Escherichia coli
Source: Antibiotics (Basel). 2024 Jun 23;13(7):581. doi: 10.3390/antibiotics13070581 (PMC11274021; doi:10.3390/antibiotics13070581)
Supplement: Supplementary file 1 [file antibiotics-13-00581-s001.zip › antibiotics-3040127-supplementary.pdf]

**Table S1**. Efficiency of plating (EOP) of *Escherichia* phage KIT06 on various *E. coli* strains. The EOP was calculated using the reference strain, *E. coli* NBRC 3972.

| *E. coli* | EOP |
|---|---|
| NBRC 3972 | 1 |
| NBRC 12062 | 4.12 |
| NBRC 13168 | 3.75 |
| NBRC 14129 | 0.33 |
| ATCC 43888 | 0 |
| ATCC 19110 | 0 |
| ATCC 23506 | 0 |
| BL21 | 3.92 |
| ME9062 | 0.61 |
| ATCC 700609 | 0.4 |
| ATCC 700891 | 0 |
| BAA-2469 | 0 |

**Table S2**. Keio collection of single-gene knockout strains used in this study related to lipopolysaccharide (LPS) synthesis in the *E. coli* K12 BW25113 strain background. The LPS structure is taken from the website of the National BioResource Project (https://shigen.nig.ac.jp/). The efficiency of plating (EOP) of *Escherichia* phage KIT06 was determined using BW25113 as the reference strain.

| Strains | Code | LPS structure | EOP |
|---|---|---|---|
| BW25113 | ME9062 | K12 LPS type and O-antigen deficient | 1 |
| Δ*waaO* | JW3602-KC | Inner core OS + Gal1-6Glc I | 0.45 |
| Δ*waaG* | JW3606-KC | Inner core OS and decreased inner core phosphorylation (phosphate groups deficient of Hep II and decreased in Hep I) | 0.9 |
| Δ*waaQ* | JW3607-KC | Core OS, Hep III deficient, and unphosphorylated Hep II (phosphate group deficient of Hep II) | 1.12 |
| Δ*waaB* | JW3603-KC | Core OS and Kdo III deficient | 0.98 |
| Δ*waaZ* | JW3599-KC | Core OS and Gal-deficient | 0.93 |

| | | | |
|---|---|---|---|
| Δ*waaC* | JW3596-KC | Truncated inner core OS; Hep I, Hep II, and Hep III deficient | 0.08 |
| Δ*waaP* | JW3605-KC | Core OS, Hep III deficient, and unphosphorylated core (phosphate groups deficient of Hep I and Hep II) | 0.89 |
| Δ*waaR* | JW3601-KC | Inner core OS + Gal1-6Glc I +Glc II | 0.59 |
| Δ*waaY* | JW3600-KC | Core OS and unphosphorylated Hep II (phosphate group deficient of Hep II) | 1.19 |
| Δ*waaF* | JW3595-KC | Truncated inner core OS; Hep II and Hep III deficient | 1.35 |

Gal, galactose; Glc, glucose; Hep, heptose; Kdo, 3-deoxy-D-manno-2-octulosonic acid; OS, oligosaccharide

**Table S3**. Keio collection of single-gene knockout strains related to outer membrane proteins or flagella proteins in the *E. coli* K12 BW25113 strain background used in this study. The efficiency of plating (EOP) of *Escherichia* phage KIT06 was determined using BW25113 strain as the reference strain.

| Strains | Code | Knockout gene | EOP |
|---|---|---|---|
| BW25113 | ME9062 | | 1 |
| Δ*ompA* | JW0940-KC | *Outer membrane protein A* | 0.72 |
| Δ*ompC* | JW2203-KC | *Outer membrane protein C* | 0.64 |
| Δ*ompF* | JW0912-KC | *Outer membrane protein F* | 0.72 |
| Δ*fepA* | JW5086-KC | *Ferrienterobactin receptor* | 0.89 |
| Δ*fhuA* | JW0146-KC | *Ferrochrome outer membrane transporter* | 0.72 |
| Δ*tonB* | JW5195-KC | *Protein Ton B* | 1.25 |
| Δ*tolC* | JW5503-KC | *Outer membrane protein Tol C* | 0.89 |
| ΔfimD | JW5780-KC | *Type I fimbriae usher protein* | 0.64 |
| Δ*fimA* | JW4277-KC | *Type 1 fimbrial protein, A chain* | 0.83 |
| Δ*fliK* | JW1927-KC | *Flagellar hook-length control protein* | 0.72 |
| Δ*fliC* | JW1908-KC | *Flagellin* | 0.72 |
| Δ*tsx* | JW0401-KC | *Nucleoside-specific channel-forming protein tsx* | 0 |
| Δ*fadL* | JW2341-KC | *Long-chain fatty acid transport protein* | 0.72 |
| Δ*lamB* | JW3996-KC | *Maltoporin* | 0.72 |
| Δ*yncD* | JW1446-KC | *Probable TonB-dependent receptor yncD* | 0.46 |

**Table S4**. ME collection gene mutants used in this study, btuB protein mutants – vitamin B12 transporter. The efficiency of plating (EOP) of *Escherichia* phage KIT06 was determined using BW25113 strain as the reference strain.

| Strains | Code | Knockout gene | EOP |
|---|---|---|---|
| BW25113 | ME9062 | *rrnB DElacZ4787 HsdR514 DE(araBAD)567 DE(rhaBAD)568 rph-1* | 1 |
| RK4784 | ME8305 | *DE(argF-lac)U169 araD139 recA1 rpsL150 flbB5301 deoC1 thi gyrA219 non metE70 DE(btuB) DE(ompC) zeh::Tn10* | 0.22 |
| RK4784 | ME 8307 | *DE(argF-lac)U169 araD139 recA1 rpsL150 fibB5301 deoC1 thi ptsF25 gyrA219 non metE70 DE(btuB) DE(ompA) zcb::Tn10* | 0.72 |
| RK4792 | 8308 ME | *DE(argF-lac)U169 araD139 relA1 rpsL150 flbB5301 deoC1 thi gyrA219 non metE70 DE(btuB) ompR151malP::Tn10* | 0.04 |

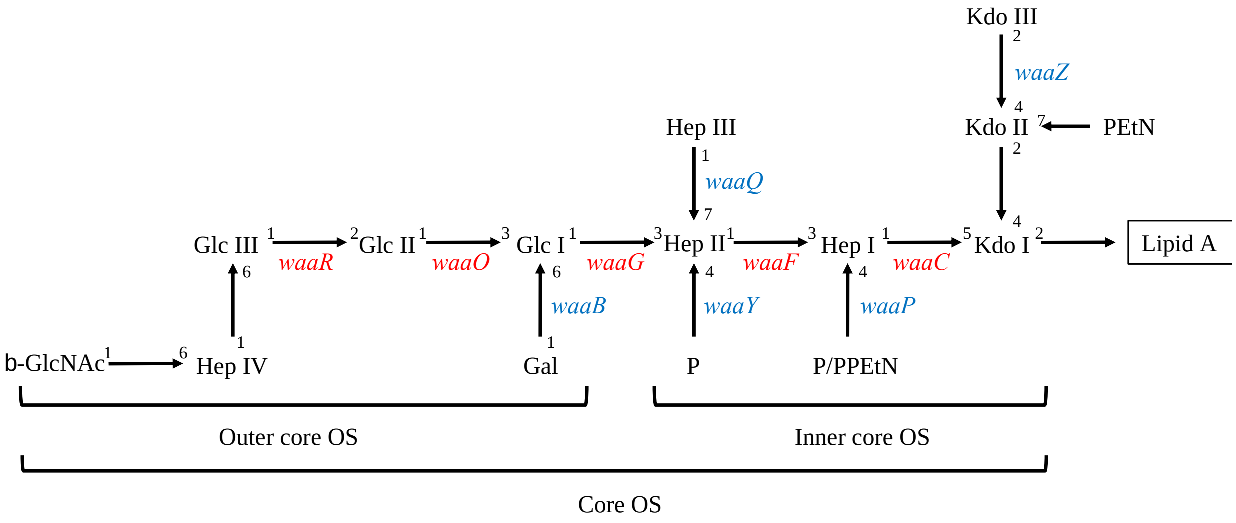


**Figure S1**. Structure of the lipopolysaccharide (LPS) core oligosaccharide (OS) of *E. coli* K-12, glycosyltransferases genes (red), and core modification-related genes (blue) responsible for core OS synthesis. Gal, Galactose; Glc, Glucose; GlcN, Glucosamine; Hep, L-glycerol-D-manno-heptose; Kdo, 3-deoxy-D-manno-2-octulosonic acid; P, phosphate; PPEtN, pyrophosphoethanolamine.

**Table S5**. Phage KIT06 genome annotation, the function of 264 ORFs, and 10 tRNAs.

| ORFs | start | stop | strand | Annotation function | |
|---|---|---|---|---|---|
| 1 | 2178 | 1 | - | rIIA lysis inhibitor | Nucleotide regulation |
| 2 | 2392 | 2189 | - | hypothetical protein | |

| | | | | | |
|---|---|---|---|---|---|
| 3 | 4264 | 2447 | - | DNA topoisomerase large subunit | Nucleotide regulation |
| 4 | 4594 | 4334 | - | hypothetical protein | |
| 5 | 4971 | 4600 | - | hypothetical protein | |
| 6 | 5150 | 4974 | - | hypothetical protein | |
| 7 | 5557 | 5153 | - | hypothetical protein | |
| 8 | 5772 | 5557 | - | cef modifier of supressor tRNAs | Nucleotide regulation |
| 9 | 6055 | 5786 | - | hypothetical protein | |
| 10 | 6644 | 6153 | - | MotB-like transcriptional regulator | Nucleotide regulation |
| 11 | 7263 | 6721 | - | hypothetical protein | |
| 12 | 7766 | 7266 | - | hypothetical protein | |
| 13 | 8513 | 7830 | - | exonuclease | Nucleotide regulation |
| 14 | 8755 | 8513 | - | hypothetical protein | |
| 15 | 8993 | 8748 | - | hypothetical protein | |
| 16 | 10334 | 9015 | - | DNA helicase | Nucleotide regulation |
| 17 | 10642 | 10331 | - | hypothetical protein | |
| 18 | 11390 | 10644 | - | putative anti-sigma factor | Nucleotide regulation |
| 19 | 12109 | 11507 | - | RNA polymerase ADP-ribosylase | Nucleotide regulation |
| 20 | 12729 | 12106 | - | RNA polymerase ADP-ribosylase | Nucleotide regulation |
| 21 | 12979 | 12797 | - | hypothetical protein | |
| 22 | 13458 | 12988 | - | molybdenum ABC transporter | Nucleotide regulation |
| 23 | 13615 | 13451 | - | hypothetical protein | |
| 24 | 13815 | 13612 | - | hypothetical protein | |
| 25 | 14275 | 13790 | - | hypothetical protein | |
| 26 | 14625 | 14284 | - | hypothetical protein | |
| 27 | 14837 | 14625 | - | hypothetical protein | |
| 28 | 15193 | 14936 | - | virion structural protein | Virion protein |
| 29 | 15419 | 15210 | - | head protein | Virion protein |
| 30 | 15625 | 15416 | - | putative small outer capsid protein | Virion protein |
| 31 | 16143 | 15625 | - | hypothetical protein | |
| 32 | 16217 | 16417 | + | hypothetical protein | |
| 33 | 17442 | 16414 | - | DNA primase | Nucleotide regulation |
| 34 | 17609 | 17445 | - | hypothetical protein | |
| 35 | 18237 | 17611 | - | hypothetical protein | |
| 36 | 18530 | 18237 | - | spackle periplasmic | Lysis |
| 37 | 18843 | 18589 | - | hypothetical protein | |
| 38 | 19149 | 18907 | - | hypothetical protein | |

| | | | | | |
|---|---|---|---|---|---|
| 39 | 19333 | 19151 | - | putative discriminator of mRNA degradation | Nucleotide regulation |
| 40 | 20819 | 19392 | - | DnaB-like replicative helicase | Nucleotide regulation |
| 41 | 21173 | 20829 | - | putative head vertex assembly chaperone | Nucleotide regulation |
| 42 | 22347 | 21166 | - | putative RecA-like recombination protein | Nucleotide regulation |
| 43 | 23267 | 22425 | - | beta-glucosyl-HMC-alpha-glucosyltransferase | Nucleotide regulation |
| 44 | 23905 | 23264 | - | hypothetical protein | |
| 45 | 24636 | 23896 | - | deoxycytidylate 5-hydroxymethyltransferase | Nucleotide regulation |
| 46 | 25041 | 24790 | - | immunity to superinfection membrane protein | Nucleotide regulation |
| 47 | 25429 | 25049 | - | hypothetical protein | |
| 48 | 28307 | 25611 | - | DNA polymerase | Nucleotide regulation |
| 49 | 28607 | 28386 | - | hypothetical protein | |
| 50 | 28977 | 28609 | - | translation repressor | Nucleotide regulation |
| 51 | 29542 | 28979 | - | clamp loader of DNA polymerase | Nucleotide regulation |
| 52 | 30503 | 29544 | - | putative replication factor C small subunit | Nucleotide regulation |
| 53 | 31241 | 30555 | - | sliding clamp DNA polymerase accessory protein | Nucleotide regulation |
| 54 | 31686 | 31297 | - | RNA polymerase binding | Nucleotide regulation |
| 55 | 31884 | 31696 | - | hypothetical protein | |
| 56 | 33580 | 31940 | - | SbcC-like subunit of palindrome specific endonuclease | Nucleotide regulation |
| 57 | 33825 | 33619 | - | hypothetical protein | |
| 58 | 34069 | 33806 | - | hypothetical protein | |
| 59 | 35085 | 34066 | - | SbcD-like subunit of palindrome specific endonuclease | Nucleotide regulation |
| 60 | 36464 | 35262 | - | alpha-glucosyltransferase | Nucleotide regulation |
| 61 | 36722 | 36531 | - | hypothetical protein | |
| 62 | 37196 | 36879 | - | hypothetical protein | |
| 63 | 37386 | 37198 | - | hypothetical protein | |
| 64 | 37957 | 37400 | - | RNA polymerase sigma factor | Nucleotide regulation |
| 65 | 38307 | 38038 | - | hypothetical protein | |
| 66 | 38519 | 38304 | - | hypothetical protein | |
| 67 | 38848 | 38522 | - | hypothetical protein | |
| 68 | 39101 | 38901 | - | hypothetical protein | |

| | | | | | |
|---|---|---|---|---|---|
| 69 | 39233 | 39102 | - | hypothetical protein | |
| 70 | 39534 | 39241 | - | hypothetical protein | |
| 71 | 39703 | 39527 | - | hypothetical protein | |
| 72 | 40185 | 39862 | - | glutaredoxin | Nucleotide regulation |
| 73 | 40468 | 40157 | - | hypothetical protein | |
| 74 | 40686 | 40471 | - | hypothetical protein | |
| 75 | 40808 | 40695 | - | hypothetical protein | |
| 76 | 41271 | 40801 | - | anaerobic ribonucleoside-triphosphate reductase activating protein | Nucleotide regulation |
| 77 | 42098 | 41268 | - | homing endonuclease | Nucleotide regulation |
| 78 | 43908 | 42091 | - | anaerobic ribonucleoside-triphosphate reductase | Nucleotide regulation |
| 79 | 44378 | 43905 | - | endonuclease VII | Nucleotide regulation |
| 80 | 44874 | 44428 | - | inhibitor of host Lon protease | Nucleotide regulation |
| 81 | 45318 | 44998 | - | ribonucleotide reductase | Nucleotide regulation |
| 82 | 45464 | 45330 | - | hypothetical protein | |
| 83 | 45718 | 45503 | - | hypothetical protein | |
| 84 | 45978 | 45715 | - | thioredoxin | Nucleotide regulation |
| 85 | 46222 | 45980 | - | hypothetical protein | |
| 86 | 46526 | 46209 | - | hypothetical protein | |
| 87 | 47452 | 46523 | - | hypothetical protein | |
| 88 | 48536 | 47505 | - | hypothetical protein | |
| 89 | 49592 | 48564 | - | hypothetical protein | |
| 90 | 50491 | 49601 | - | hypothetical protein | |
| 91 | 50906 | 50499 | - | hypothetical protein | |
| 92 | 51489 | 50962 | - | putative thioredoxin | Nucleotide regulation |
| 93 | 51852 | 51550 | - | hypothetical protein | |
| 94 | 52922 | 51954 | - | putative thioredoxin | Nucleotide regulation |
| 95 | 53255 | 52992 | - | hypothetical protein | |
| 96 | 53401 | 53252 | - | hypothetical protein | |
| 97 | 54524 | 53514 | - | hypothetical protein | |
| 98 | 54985 | 54524 | - | hypothetical protein | |
| 99 | 55509 | 54988 | - | hypothetical protein | |
| 100 | 56046 | 55516 | - | hypothetical protein | |
| 101 | 56314 | 56048 | - | hypothetical protein | |
| 102 | 56713 | 56540 | - | hypothetical protein | |
| 103 | 56897 | 56703 | - | hypothetical protein | |
| 104 | 57103 | 56900 | - | hypothetical protein | |
| 105 | 57291 | 57103 | - | hypothetical protein | |

| | | | | | |
|---|---|---|---|---|---|
| 106 | 57773 | 57387 | - | starvation-inducible transcriptional regulator | Nucleotide regulation |
| 107 | 58063 | 57770 | - | lysis inhibition | Lysis |
| 108 | 58288 | 58076 | - | hypothetical protein | |
| 109 | 58912 | 58331 | - | thymidine kinase | Nucleotide regulation |
| 110 | 59107 | 58922 | - | hypothetical protein | |
| 111 | 59316 | 59104 | - | hypothetical protein | |
| 112 | 59767 | 59288 | - | phosphatase | Nucleotide regulation |
| 113 | 60105 | 59764 | - | valyl tRNA synthetase modifier | Nucleotide regulation |
| 114 | 60643 | 60098 | - | hypothetical protein | |
| 115 | 61112 | 60651 | - | endoribonuclease | Nucleotide regulation |
| 116 | 61450 | 61172 | - | hypothetical protein | |
| 117 | 61716 | 61450 | - | hypothetical protein | |
| 118 | 61930 | 61709 | - | hypothetical protein | |
| 119 | 62292 | 61930 | - | autonomous glycyl radical cofactor | Nucleotide regulation |
| 120 | 62630 | 62301 | - | hypothetical protein | |
| 121 | 63166 | 62627 | - | hypothetical protein | |
| 122 | 63922 | 63344 | - | internal protein III | Virion protein |
| 123 | 64266 | 63949 | - | hypothetical protein | |
| 124 | 64832 | 64338 | - | endolysin | Lysis |
| 125 | 65309 | 64869 | - | nudix hydrolase | Nucleotide regulation |
| 126 | 65794 | 65306 | - | hypothetical protein | |
| 127 | 66165 | 65791 | - | hypothetical protein | |
| 128 | 67116 | 66508 | - | hypothetical protein | |
| 129 | 67751 | 67158 | - | hypothetical protein | |
| 130 | 67971 | 67795 | - | hypothetical protein | |
| 131 | 68303 | 68040 | - | hypothetical protein | |
| 132 | 69200 | 68850 | - | hypothetical protein | |
| | 69292 | 69217 | - | tRNA-Arg-TCT | |
| | 69371 | 69297 | - | tRNA-Asn-GTT | |
| | 69462 | 69376 | - | tRNA-Tyr-GTA | |
| | 69546 | 69472 | - | tRNA-Met-CAT | |
| | 69623 | 69548 | - | tRNA-Thr-TGT | |
| | 69718 | 69629 | - | tRNA-Ser-TGA | |
| | 69794 | 69721 | - | tRNA-Pro-TGG | |
| | 69879 | 69805 | - | tRNA-Gly-TCC | |
| | 69971 | 69885 | - | tRNA-Leu-TAA | |
| | 70045 | 69973 | - | tRNA-Gln-TTG | |
| 133 | 70647 | 70132 | - | hypothetical protein | |
| 134 | 70940 | 70650 | - | hypothetical protein | |
| 135 | 71347 | 70943 | - | hypothetical protein | |

| | | | | | |
|---|---|---|---|---|---|
| 136 | 71533 | 71348 | - | hypothetical protein | |
| 137 | 71818 | 71690 | - | hypothetical protein | |
| 138 | 72195 | 71887 | - | hypothetical protein | |
| 139 | 72528 | 72256 | - | hypothetical protein | |
| 140 | 72892 | 72605 | - | hypothetical protein | |
| 141 | 73420 | 72965 | - | hypothetical protein | |
| 142 | 73662 | 73420 | - | hypothetical protein | |
| 143 | 74375 | 73662 | - | tail fiber chaperone | Nucleotide regulation |
| 144 | 74955 | 74425 | - | deoxynucleoside monophosphate kinase | Nucleotide regulation |
| 145 | 75886 | 75062 | - | tail completion protein | Virion protein |
| 146 | 76338 | 75886 | - | DNA end protector | Virion protein |
| 147 | 76386 | 76976 | + | head completion protein | Virion protein |
| 148 | 76960 | 78687 | + | baseplate wedge subunit | Virion protein |
| 149 | 78722 | 79216 | + | baseplate hub subunit and tail lysozyme | Virion protein |
| 150 | 79217 | 79510 | + | hypothetical protein | |
| 151 | 79519 | 81501 | + | PAAR motif of membrane proteins | Virion protein |
| 152 | 81498 | 83816 | + | baseplate wedge subunit | Virion protein |
| 153 | 83770 | 84723 | + | phage baseplate wedge initiator | Virion protein |
| 154 | 84716 | 85720 | + | baseplate wedge subunit | Virion protein |
| 155 | 85784 | 86650 | + | putative baseplate wedge subunit | Virion protein |
| 156 | 86650 | 88458 | + | baseplate wedge tail fiber protein connector | Virion protein |
| 157 | 88458 | 89117 | + | baseplate wedge protein | Virion protein |
| 158 | 89114 | 90697 | + | baseplate wedge subunit | Virion protein |
| 159 | 90694 | 92157 | + | tail collar fiber protein | Virion protein |
| 160 | 92190 | 93119 | + | putative fibritin neck whiskers protein | Virion protein |
| 161 | 93121 | 93891 | + | head-tail adaptor Ad2 | Virion protein |
| 162 | 93933 | 94751 | + | neck protein | Virion protein |
| 163 | 94760 | 95254 | + | tail sheath stabilizer | Virion protein |
| 164 | 95238 | 97070 | + | terminase small subunit | Nucleotide regulation |
| 165 | 97102 | 99081 | + | terminase large subunit | Nucleotide regulation |
| 166 | 99198 | 99689 | + | tail sheath | Virion protein |
| 167 | 99773 | 101347 | + | tail protein | Virion protein |
| 168 | 101347 | 101580 | + | portal protein | Virion protein |
| 169 | 101580 | 102005 | + | prohead core protein | Virion protein |
| 170 | 102005 | 102643 | + | head scaffolding protein | Virion protein |
| 171 | 102674 | 103483 | + | head maturation protease | Virion protein |
| 172 | 103502 | 105067 | + | head scaffolding protein | Virion protein |
| 173 | 105383 | 105117 | - | major head protein | Virion protein |
| 174 | 105486 | 106769 | + | hypothetical protein | |
| 175 | 107803 | 106799 | - | head vertex subunit precursor | Virion protein |
| 176 | 108091 | 107813 | - | RNA ligase | Nucleotide regulation |

| | | | | | |
|---|---|---|---|---|---|
| 177 | 108281 | 108078 | - | hypothetical protein | |
| 178 | 109233 | 108382 | - | hypothetical protein | |
| 179 | 109923 | 109243 | - | capsid and scaffold protein | Virion protein |
| 180 | 109974 | 111485 | + | minor head protein inhibitor of protease | Virion protein |
| 181 | 111511 | 111741 | + | DNA helicase | Nucleotide regulation |
| 182 | 111964 | 111797 | - | Chain A, ATP-dependent DNA helicase uvsW | Nucleotide regulation |
| 183 | 112217 | 111993 | - | hypothetical protein | |
| 184 | 112630 | 112217 | - | hypothetical protein | |
| 185 | 113095 | 112697 | - | UvsY-like recombination mediator | Nucleotide regulation |
| 186 | 113721 | 113095 | - | baseplate wedge subunit | Virion protein |
| 187 | 113772 | 114521 | + | baseplate hub | Virion protein |
| 188 | 114521 | 115696 | + | baseplate hub | Virion protein |
| 189 | 115716 | 116174 | + | baseplate hub | Virion protein |
| 190 | 116171 | 117943 | + | baseplate distal hub subunit | Virion protein |
| 191 | 117952 | 119046 | + | baseplate tail tube cap | Virion protein |
| 192 | 119046 | 120011 | + | tail tube | Virion protein |
| 193 | 120330 | 120040 | - | hypothetical protein | |
| 194 | 122448 | 120391 | - | hypothetical protein | |
| 195 | 124545 | 122452 | - | RNA polymerase-ADP-ribosyltransferase | Nucleotide regulation |
| 196 | 124786 | 124598 | - | hypothetical protein | |
| 197 | 126243 | 124783 | - | DNA ligase | Nucleotide regulation |
| 198 | 126509 | 126243 | - | hypothetical protein | |
| 199 | 127339 | 126509 | - | hypothetical protein | |
| 200 | 127794 | 127336 | - | hypothetical protein | |
| 201 | 127993 | 127787 | - | hypothetical protein | |
| 202 | 128187 | 127990 | - | hypothetical protein | |
| 203 | 128474 | 128187 | - | hypothetical protein | |
| 204 | 128880 | 128515 | - | hypothetical protein | |
| 205 | 129281 | 128949 | - | hypothetical protein | |
| 206 | 129568 | 129392 | - | hypothetical protein | |
| 207 | 130214 | 129966 | - | lysis inhibition accessory protein | Lysis |
| 208 | 130697 | 130362 | - | head morphogenesis | Virion protein |
| 209 | 131062 | 130754 | - | SH3 beta-barrel fold-containing protein | Nucleotide regulation |
| 210 | 131299 | 131063 | - | tail fibers protein | Virion protein |
| 211 | 131880 | 131299 | - | deoxycytidylate deaminase | Nucleotide regulation |
| 212 | 132215 | 131877 | - | hypothetical protein | |
| 213 | 132448 | 132212 | - | hypothetical protein | |
| 214 | 132785 | 132510 | - | hypothetical protein | |
| 215 | 133758 | 132850 | - | putative polynucleotide kinase | Nucleotide regulation |

| | | | | | |
|---|---|---|---|---|---|
| 216 | 134057 | 133755 | - | hypothetical protein | |
| 217 | 134285 | 134109 | - | hypothetical protein | |
| 218 | 134581 | 134282 | - | outer membrane lipoprotein Rz1 | Nucleotide regulation |
| 219 | 134931 | 134578 | - | putative spanin, inner membrane subunit | Nucleotide regulation |
| 220 | 135428 | 134922 | - | inhibitor of host transcription | Nucleotide regulation |
| 221 | 136614 | 135490 | - | RNA ligase | Nucleotide regulation |
| 222 | 137077 | 136667 | - | endonuclease | Nucleotide regulation |
| 223 | 138283 | 137105 | - | ribonucleotide reductase class Ia beta subunit | Nucleotide regulation |
| 224 | 140599 | 138335 | - | NrdA-like aerobic NDP reductase large subunit | Nucleotide regulation |
| 225 | 141133 | 140870 | - | hypothetical protein | |
| 226 | 142017 | 141157 | - | thymidylate synthase | Nucleotide regulation |
| 227 | 142410 | 142063 | - | putative dihydrofolate reductase | Nucleotide regulation |
| 228 | 143012 | 142431 | - | dihydrofolate reductase | Nucleotide regulation |
| 229 | 143257 | 143012 | - | hypothetical protein | |
| 230 | 143510 | 143268 | - | hypothetical protein | |
| 231 | 143945 | 143565 | - | hypothetical protein | |
| 232 | 144217 | 143990 | - | hypothetical protein | |
| 233 | 145274 | 144366 | - | single strand DNA binding protein | Nucleotide regulation |
| 234 | 146027 | 145374 | - | DNA helicase loader | Nucleotide regulation |
| 235 | 146362 | 146024 | - | RNA polymerase-associated protein Gp33 | Nucleotide regulation |
| 236 | 146609 | 146340 | - | putative double-stranded DNA-binding protein | Nucleotide regulation |
| 237 | 147535 | 146618 | - | RNase H | Nucleotide regulation |
| 238 | 147640 | 151509 | + | long tail fiber proximal subunit | Virion protein |
| 239 | 151518 | 152633 | + | long tail fiber protein proximal connector | Virion protein |
| 240 | 152696 | 153358 | + | putative tail connector protein | Virion protein |
| 241 | 153367 | 157278 | + | long tail fiber protein distal subunit | Virion protein |
| 242 | 157309 | 158100 | + | receptor recognizing protein | Virion protein |
| 243 | 158131 | 158787 | + | holin | Lysis |
| 244 | 159060 | 158788 | - | Chain I, kDa anti-sigma factor | Nucleotide regulation |
| 245 | 159225 | 159073 | - | hypothetical protein | |

| | | | | | |
|---|---|---|---|---|---|
| 246 | 159500 | 159222 | - | inhibitor of MrcBC restriction | Nucleotide regulation |
| 247 | 159715 | 159584 | - | putative anti-restriction nuclease | Nucleotide regulation |
| 248 | 160081 | 159785 | - | hypothetical protein | |
| 249 | 160542 | 160081 | - | anti-restriction nuclease | Nucleotide regulation |
| 250 | 160868 | 160539 | - | hypothetical protein | |
| 251 | 161514 | 160879 | - | middle transcription regulatory protein | Nucleotide regulation |
| 252 | 161791 | 161642 | - | transcriptional regulator | Nucleotide regulation |
| 253 | 163116 | 161788 | - | DNA topoisomerase II | Nucleotide regulation |
| 254 | 163409 | 163254 | - | putative acridine resistance protein | Nucleotide regulation |
| 255 | 163952 | 163497 | - | Ndd-like nucleoid disruption protein | Nucleotide regulation |
| 256 | 164228 | 164013 | - | hypothetical protein | |
| 257 | 164347 | 164237 | - | hypothetical protein | |
| 258 | 164541 | 164344 | - | hypothetical protein | |
| 259 | 164662 | 164549 | - | hypothetical protein | |
| 260 | 164826 | 164728 | - | outer membrane protein | Virion protein |
| 261 | 165169 | 164906 | - | hypothetical protein | |
| 262 | 165800 | 165243 | - | endonuclease | Nucleotide regulation |
| 263 | 166081 | 165887 | - | endonuclease | Nucleotide regulation |
| 264 | 167048 | 166110 | - | rIIB lysis inhibitor | Nucleotide regulation |

**Table S6.** Classification of putative ORFs in *Escherichia* phage KIT06 genome by predicted function. tRNAs are not included.

| ORF | Start | End | Strand | Predicted function | Best BLAST hit | | | |
|---|---|---|---|---|---|---|---|---|
| | | | | | Related Organism | Similarity (%) | E-value | GenBank No. |
| **Nucleotide regulation** | | | | | | | | |
| 1 | 1 | 2178 | - | rIIA lysis inhibitor | protein rIIA [Escherichia phage W143] | 98.9 | 0 | QWV60351.1 |
| 3 | 2447 | 4264 | - | DNA topoisomerase large subunit | DNA topoisomerase II large subunit [Escherichia phage vB_EcoM_IME537] | 100 | 0 | WP_171921514.1 |
| 8 | 5557 | 5772 | - | cef modifier of supressor tRNAs | cef modifier of supressor tRNAs [Escherichia phage vB_EcoM_NBG2] | 100 | 4.00E-44 | YP_010072031.1 |
| 10 | 6153 | 6644 | - | MotB-like transcriptional regulator | MotB-like transcriptional regulator [Enterobacteria phage vB_EcoM_IME339] | 100 | 5.00E-115 | YP_010094667.1 |
| 13 | 7830 | 8513 | - | exonuclease | exonuclease [Escherichia phage slur02] | 100 | 3.00E-167 | YP_009210205.1 |
| 16 | 9015 | 10334 | - | DNA helicase | Dda-like helicase [Escherichia phage vB_EcoM_ACG-C40] | 100 | 0 | YP_006986566.1 |
| 19 | 11507 | 12109 | - | RNA polymerase ADP-ribosylase | RNA polymerase ADP-ribosylase [Citrobacter phage vB_CroM_CrRp10] | 100 | 5.00E-147 | YP_010065344.1 |
| 20 | 12106 | 12729 | - | RNA polymerase ADP-ribosylase | RNA polymerase ADP-ribosylase [Escherichia phage vB_EcoM_IME537] | 99.5 | 2.00E-150 | YP_010071058.1 |
| 22 | 12988 | 13458 | - | molybdenum ABC transporter | molybdenum ABC transporter [Escherichia phage vB_EcoM_WL-3] | 99.4 | 2.00E-109 | QOQ37398.1 |
| 33 | 16414 | 17442 | - | DNA primase | DNA primase [Escherichia phage HY01] | 100 | 0 | YP_009148481.1 |
| 39 | 19151 | 19333 | - | putative discriminator of mRNA degradation | putative discriminator of mRNA degradation [Shigella phage vB_SboM_Phaginator] | 98.3 | 1.00E-34 | UGO46713.1 |
| 40 | 19392 | 20819 | - | DnaB-like replicative helicase | DnaB-like replicative helicase [Escherichia phage PE37] | 100 | 0 | YP_010073326.1 |

| | | | | | | | | |
|---|---|---|---|---|---|---|---|---|
| 41 | 20829 | 21173 | - | putative head vertex assembly chaperone | gp40 head vertex assembly chaperone [Enterobacteriaceae] | 99.1 | 1.00E-74 | WP_015969201.1 |
| 42 | 21166 | 22347 | - | putative RecA-like recombination protein | putative RecA-like recombination protein [Escherichia phage N2] | 99.8 | 0 | UTQ79362.1 |
| 43 | 22425 | 23267 | - | beta-glucosyl-HMC-alpha-glucosyltransferase | beta-glucosyl-HMC-alpha-glucosyltransferase [Escherichia phage ime09] | 99.6 | 0 | YP_007004427.1 |
| 45 | 23896 | 24636 | - | deoxycytidylate 5-hydroxymethyltransferase | deoxycytidylate 5-hydroxymethyltransferase [Escherichia phage EP01] | 99.2 | 0 | UIU46795.1 |
| 46 | 24790 | 25041 | - | immunity to superinfection membrane protein | immunity to superinfection membrane protein [Escherichia phage vB_EcoM_SYGD1] | 100 | 1.00E-49 | QUD16027.1 |
| 48 | 25611 | 28307 | - | DNA polymerase | DNA polymerase [Escherichia phage vB_EcoM_SYGD1] | 100 | 0 | QUD16025.1 |
| 50 | 28609 | 28977 | - | translation repressor | translation repressor [Shigella phage pSs-1] | 99.2 | 2.00E-84 | YP_009110868.1 |
| 51 | 28979 | 29542 | - | clamp loader of DNA polymerase | clamp loader of DNA polymerase [Escherichia phage HY01] | 99.5 | 3.00E-134 | YP_009148498.1 |
| 52 | 29544 | 30503 | - | putative replication factor C small subunit | putative replication factor C small subunit [Escherichia phage JLBYU24] | 100 | 0 | UGO55400.1 |
| 53 | 30555 | 31241 | - | sliding clamp DNA polymerase accessory protein | sliding clamp DNA polymerase accessory protein [Shigella phage ESh16] | 99.6 | 1.00E-164 | URY11627.1 |
| 54 | 31297 | 31686 | - | RNA polymerase binding | RNA polymerase binding [Escherichia phage slur02] | 100 | 8.00E-91 | YP_009210244.1 |
| 56 | 31940 | 33580 | - | SbcC-like subunit of palindrome specific endonuclease | SbcC-like subunit of palindrome specific endonuclease [Escherichia phage ime09] | 100 | 0 | YP_007004439.1 |
| 59 | 34066 | 35085 | - | SbcD-like subunit of palindrome specific endonuclease | SbcD-like subunit of palindrome specific endonuclease [Shigella phage Sf24] | 99.7 | 0 | YP_009619267.1 |

| | | | | | | | | |
|---|---|---|---|---|---|---|---|---|
| 60 | 35262 | 36464 | - | alpha-glucosyltransferase | alpha-glucosyltransferase [Shigella phage Shfl2] | 99.8 | 0 | YP_004414960.1 |
| 64 | 37400 | 37957 | - | RNA polymerase sigma factor | RNA polymerase sigma factor [Escherichia phage vB_EcoM_112] | 100 | 2.00E-135 | YP_009030673.1 |
| 72 | 39862 | 40185 | - | glutaredoxin | glutaredoxin [Escherichia phage vB_EcoM_ASO78A] | 99.1 | 1.00E-71 | UAW58507.1 |
| 76 | 40801 | 41271 | - | anaerobic ribonucleoside-triphosphate reductase activating protein | anaerobic ribonucleoside-triphosphate reductase activating protein [Escherichia phage Ec_MI-02] | 99.4 | 4.00E-111 | UZV41401.1 |
| 77 | 41268 | 42098 | - | homing endonuclease | homing endonuclease [Escherichia phage EC121] | 100 | 0 | YP_010067764.1 |
| 78 | 42091 | 43908 | - | anaerobic ribonucleoside-triphosphate reductase | anaerobic ribonucleoside reductase large subunit [Escherichia phage EC121] | 99.3 | 0 | YP_010067765.1 |
| 79 | 43905 | 44378 | - | endonuclease VII | endonuclease VII [Escherichia phage vB_EcoM_G9062] | 99.4 | 1.00E-111 | YP_010070733.1 |
| 80 | 44428 | 44874 | - | inhibitor of host Lon protease | inhibitor of host Lon protease [Escherichia phage REP4] | 98.7 | 3.00E-103 | WBY53205.1 |
| 81 | 44998 | 45318 | - | ribonucleotide reductase | ribonucleotide reductase [Escherichia phage vB_EcoM_G50] | 97.2 | 4.00E-71 | YP_010069912.1 |
| 84 | 45715 | 45978 | - | thioredoxin | phage-associated thioredoxin [Escherichia phage ime09] | 100 | 9.00E-58 | YP_007004467.1 |
| 92 | 50962 | 51489 | - | putative thioredoxin | putative thioredoxin [Escherichia phage JLBYU24] | 100 | 3.00E-122 | UGO55440.1 |
| 94 | 51954 | 52922 | - | putative thioredoxin | putative thioredoxin [Shigella phage vB_SboM_Phaginator] | 99.7 | 0 | UGO46768.1 |
| 106 | 57387 | 57773 | - | starvation-inducible transcriptional regulator | starvation-inducible transcriptional regulator [Escherichia phage wV7] | 99.2 | 1.00E-86 | YP_007004849.1 |
| 109 | 58331 | 58912 | - | thymidine kinase | thymidine kinase [Escherichia phage L14] | 99.5 | 4.00E-141 | WNV49251.1 |
| 112 | 59288 | 59767 | - | phosphatase | phosphatase [Escherichia phage ime09] | 100 | 3.00E-113 | YP_007004492.1 |

| | | | | | | | | |
|---|---|---|---|---|---|---|---|---|
| 113 | 59764 | 60105 | - | valyl tRNA synthetase modifier | valyl tRNA synthetase modifier [Salmonella phage pSe_SNUABM_01] | 100 | 5.00E-76 | YP_010075380.1 |
| 115 | 60651 | 61112 | - | endoribonuclease | endoribonuclease [Escherichia phage vB_VIPECOOM01] | 99.4 | 2.00E-109 | WFG77737.1 |
| 119 | 61930 | 62292 | - | autonomous glycyl radical cofactor | autonomous glycyl radical cofactor [Shigella phage ESh28] | 99.2 | 1.00E-81 | URY14129.1 |
| 125 | 64869 | 65309 | - | nudix hydrolase | nudix hydrolase [Escherichia phage EP01] | 100 | 2.00E-106 | UIU46874.1 |
| 143 | 73662 | 74375 | - | tail fiber chaperone | tail fiber chaperone [Escherichia phage vB_EcoM_Ozark] | 97.5 | 8.00E-45 | YP_010072708.1 |
| 144 | 74425 | 74955 | - | deoxynucleoside monophosphate kinase | deoxynucleoside monophosphate kinase [Escherichia phage HY03] | 100 | 8.00E-175 | YP_009284004.1 |
| 164 | 97070 | 95238 | + | terminase small subunit | terminase small subunit [Shigella phage Sf24] | 99.4 | 1.00E-116 | YP_009619098.1 |
| 165 | 99081 | 97102 | + | terminase large subunit | terminase large subunit [Escherichia phage slur02] | 99.8 | 0 | YP_009210351.1 |
| 176 | 107813 | 108091 | - | RNA ligase | RNA ligase [Escherichia phage vB_EcoM_IME537] | 99.7 | 0 | YP_010070944.1 |
| 181 | 111741 | 111511 | + | DNA helicase | DNA helicase [Escherichia phage vB_EcoM_IME537] | 99.8 | 0 | YP_010070950.1 |
| 182 | 111797 | 111964 | - | Chain A, ATP-dependent DNA helicase uvsW | DNA helicase [Escherichia phage vB_EcoM_112] | 100 | 1.00E-44 | YP_009030789.1 |
| 185 | 112697 | 113095 | - | UvsY-like recombination mediator | UvsY-like recombination mediator [Escherichia phage T4] | 98.5 | 1.00E-92 | NP_049799.2 |
| 195 | 122452 | 124545 | - | RNA polymerase-ADP-ribosyltransferase | RNA polymerase-ADP-ribosyltransferase [Escherichia phage EP01] | 99.7 | 0 | UIU46945.1 |
| 197 | 124783 | 126243 | - | DNA ligase | DNA ligase [Shigella phage Sf21] | 99.8 | 0 | YP_009619006.1 |
| 209 | 130754 | 131062 | - | SH3 beta-barrel fold-containing protein | SH3 beta-barrel fold-containing protein [Shigella phage Shfl2] | 100 | 1.00E-66 | YP_004415104.1 |

| | | | | | | | | |
|---|---|---|---|---|---|---|---|---|
| 211 | 131299 | 131880 | - | deoxycytidylate deaminase | deoxycytidylate deaminase [Escherichia phage MLP2] | 98.5 | 4.00E-139 | UEN68680.1 |
| 215 | 132850 | 133758 | - | putative polynucleotide kinase | putative polynucleotide kinase [Escherichia phage vB_EcoM_R5505] | 98.7 | 0 | QBQ79832.1 |
| 218 | 134282 | 134581 | - | outer membrane lipoprotein Rz1 | outer membrane lipoprotein Rz1 [Escherichia phage GADU22] | 99 | 3.00E-66 | WIK99951.1 |
| 219 | 134578 | 134931 | - | putative spanin, inner membrane subunit | putative spanin, inner membrane subunit [Escherichia phage 132] | 99.2 | 7.00E-77 | QWY90593.1 |
| 220 | 134922 | 135428 | - | inhibitor of host transcription | inhibitor of host transcription [Escherichia phage vB_EcoM_IME537] | 100 | 1.00E-121 | YP_010070993.1 |
| 221 | 135490 | 136614 | - | RNA ligase | RNA ligase [Escherichia phage BYEP01] | 99.7 | 0 | WPK17811.1 |
| 222 | 136667 | 137077 | - | endonuclease | endonuclease [Enterobacteria phage Aplg8] | 100 | 1.00E-94 | YP_010066017.1 |
| 223 | 137105 | 138283 | - | ribonucleotide reductase class Ia beta subunit | ribonucleotide reductase class Ia beta subunit [Escherichia phage vB_EcoM-G28] | 100 | 0 | YP_010069789.1 |
| 224 | 138335 | 140599 | - | NrdA-like aerobic NDP reductase large subunit | NrdA-like aerobic NDP reductase large subunit [Enterobacteria phage vB_EcoM_IME340] | 100 | 0 | YP_010066426.1 |
| 226 | 141157 | 142017 | - | thymidylate synthase | thymidylate synthase [Enterobacteria phage LZ2] | 99.7 | 0 | AAP86751.1 |
| 227 | 142063 | 142410 | - | putative dihydrofolate reductase | putative dihydrofolate reductase [Escherichia phage JLBYU22] | 98.3 | 3.00E-79 | UGO56576.1 |
| 228 | 142431 | 143012 | - | dihydrofolate reductase | dihydrofolate reductase [Escherichia phage ECO07P1] | 99.5 | 4.00E-141 | WAX12840.1 |
| 233 | 144366 | 145274 | - | single strand DNA binding protein | single strand DNA binding protein [Escherichia phage EC121] | 100 | 0 | YP_010067922.1 |
| 234 | 145374 | 146027 | - | DNA helicase loader | DNA helicase loader [Escherichia phage ime09] | 99.5 | 3.00E-154 | YP_007004615.1 |

| | | | | | | | | |
|---|---|---|---|---|---|---|---|---|
| 235 | 146024 | 146362 | - | RNA polymerase-associated protein Gp33 | RNA polymerase-associated protein Gp33 [Escherichia phage AUBRB02] | 99.1 | 6.00E-73 | CAK6624447.1 |
| 236 | 146340 | 146609 | - | putative double-stranded DNA-binding protein | putative double-stranded DNA-binding protein [Escherichia phage U115] | 98.9 | 4.00E-55 | UAV89206.1 |
| 237 | 146618 | 147535 | - | RNase H | RNase H [Escherichia phage vB_EcoM_IME537] | 100 | 0 | YP_010071013.1 |
| 244 | 158788 | 159060 | - | Chain I, kDa anti-sigma factor | anti-sigma factor [Enterobacteria phage RB18] | 98.9 | 2.00E-56 | YP_010067133.1 |
| 246 | 159222 | 159500 | - | inhibitor of MrcBC restriction | inhibitor of MrcBC restriction [Escherichia phage HY03] | 100 | 6.00E-58 | YP_009284205.1 |
| 247 | 159584 | 159715 | - | putative anti-restriction nuclease | putative anti-restriction nuclease [Escherichia phage JLBYU24] | 100 | 2.00E-23 | UGO55601.1 |
| 249 | 160081 | 160542 | - | anti-restriction nuclease | anti-restriction nuclease [Escherichia phage EcNP1] | 98 | 3.00E-108 | YP_010068162.1 |
| 251 | 160879 | 161514 | - | middle transcription regulatory protein | middle transcription regulatory protein [Escherichia phage BW-1] | 99.5 | 8.00E-150 | WKV22920.1 |
| 252 | 161642 | 161791 | - | transcriptional regulator | transcriptional regulator [Enterobacteria phage GiZh] | 98 | 6.00E-22 | YP_010066207.1 |
| 253 | 161788 | 163116 | - | DNA topoisomerase II | DNA topoisomerase II [Escherichia phage EC121] | 99.8 | 0 | YP_010067942.1 |
| 254 | 163254 | 163409 | - | putative acridine resistance protein | putative acridine resistance protein [Escherichia phage HY03] | 96.1 | 4.00E-24 | YP_009284198.1 |
| 255 | 163497 | 163952 | - | Ndd-like nucleoid disruption protein | Ndd-like nucleoid disruption protein [Shigella phage vb_GEC_SH_GT] | 99.3 | 3.00E-108 | WRN92411.1 |
| 262 | 165243 | 165800 | - | endonuclease | endonuclease [Shigella phage ESh31] | 99.5 | 5.00E-136 | URY15099.1 |
| 263 | 165887 | 166081 | - | endonuclease | Phage endonuclease (ACLAME 960) [Escherichia phage T4_ev240] | 98.4 | 4.00E-36 | VUF56078.1 |
| 264 | 166110 | 167048 | - | rIIB lysis inhibitor | RIIB lysis inhibitor [Escherichia phage slur02] | 99.4 | 0 | YP_009210192.1 |
| **Virion proteins** | | | | | | | | |

| | | | | | | | | |
|---|---|---|---|---|---|---|---|---|
| 28 | 14936 | 15193 | - | virion structural protein | virion structural protein [Enterobacteria phage RB27] | 100 | 1.00E-55 | YP_009102235.1 |
| 29 | 15210 | 15419 | - | head protein | head protein [Escherichia phage slur14] | 100 | 6.00E-43 | YP_009180802.1 |
| 30 | 15416 | 15625 | - | putative small outer capsid protein | putative small outer capsid protein [Escherichia phage vB_EcoM_Nami] | 98.6 | 2.00E-39 | QXV73485.1 |
| 122 | 63344 | 63922 | - | internal protein III | internal protein III [Escherichia phage ECO07P1] | 98.4 | 4.00E-133 | WAX12950.1 |
| 145 | 75062 | 75886 | - | tail completion protein | tail completion protein [Salmonella phage GRNsp7] | 99.4 | 5.00E-126 | USW07316.1 |
| 146 | 75886 | 76338 | - | DNA end protector | DNA end protector [Escherichia phage HY01] | 100 | 0 | YP_009148588.1 |
| 147 | 76976 | 76386 | + | head completion protein | head completion protein [Escherichia phage JEP6] | 99.3 | 7.00E-106 | QOC55343.1 |
| 148 | 78687 | 76960 | + | baseplate wedge subunit | baseplate wedge subunit [Enterobacteria phage vB_EcoM_IME339] | 99.5 | 3.00E-141 | YP_010094538.1 |
| 149 | 79216 | 78722 | + | baseplate hub subunit and tail lysozyme | baseplate hub subunit and tail lysozyme [Yersinia phage fPS-90] | 99.8 | 0 | YP_010091185.1 |
| 151 | 81501 | 79519 | + | PAAR motif of membrane proteins | PAAR motif of membran proteins [Escherichia phage vB_EcoM-G28] | 99.8 | 8.00E-64 | YP_010069712.1 |
| 152 | 83816 | 81498 | + | baseplate wedge subunit | baseplate wedge subunit [Citrobacter phage vB_CroM_CrRp10] | 100 | 0 | YP_010065213.1 |
| 153 | 84723 | 83770 | + | phage baseplate wedge initiator | Phage baseplate wedge initiator (T4-like gp7) [Escherichia phage vB_Eco_Solly] | 99.7 | 0 | CAJ1578533.1 |
| 154 | 85720 | 84716 | + | baseplate wedge subunit | baseplate wedge subunit [Escherichia phage slur02] | 96.1 | 0 | YP_009210340.1 |
| 155 | 86650 | 85784 | + | putative baseplate wedge subunit | putative baseplate wedge subunit [Escherichia phage vB_EcoM_R5505] | 99.7 | 0 | QBQ79767.1 |
| 156 | 88458 | 86650 | + | baseplate wedge tail fiber protein connector | baseplate wedge tail fiber protein connector [Escherichia phage EC121] | 99.7 | 0 | YP_010067837.1 |

| | | | | | | | | |
|---|---|---|---|---|---|---|---|---|
| 157 | 89117 | 88458 | + | baseplate wedge protein | baseplate wedge protein [Escherichia phage 310Ecol104PP] | 99.8 | 0 | WLY86346.1 |
| 158 | 90697 | 89114 | + | baseplate wedge subunit | baseplate wedge subunit [Shigella phage vb_GEC_SH_GT] | 99.5 | 8.00E-158 | WRN92576.1 |
| 159 | 92157 | 90694 | + | tail collar fiber protein | tail collar fiber protein [Escherichia phage REP4] | 99.6 | 0 | WBY53280.1 |
| 160 | 93119 | 92190 | + | putative fibritin neck whiskers protein | putative fibritin neck whiskers protein [Escherichia phage JLBYU24] | 99.6 | 0 | UGO55506.1 |
| 161 | 93891 | 93121 | + | head-tail adaptor Ad2 | head-tail adaptor Ad2 [Shigella phage SH7] | 100 | 0 | YP_010076777.1 |
| 162 | 94751 | 93933 | + | neck protein | neck protein [Escherichia phage BF15] | 100 | 0 | QXN75864.1 |
| 163 | 95254 | 94760 | + | tail sheath stabilizer | tail sheath stabilizer [Shigella phage Shfl2] | 99.6 | 0 | YP_004415057.1 |
| 166 | 99689 | 99198 | + | tail sheath | tail sheath [Escherichia phage RB32] | 99.7 | 0 | YP_803108.1 |
| 167 | 101347 | 99773 | + | tail protein | tail protein [Escherichia phage ECML-134] | 100 | 6.00E-117 | YP_009102639.1 |
| 168 | 101580 | 101347 | + | portal protein | portal protein [Yersinia phage fPS-2] | 99.8 | 0 | YP_010077052.1 |
| 169 | 102005 | 101580 | + | prohead core protein | prohead [Shigella phage Shfl2] | 98.7 | 1.00E-40 | YP_004415063.1 |
| 170 | 102643 | 102005 | + | head scaffolding protein | head scaffolding protein [Salmonella phage SG1] | 99.3 | 2.00E-95 | YP_010075065.1 |
| 171 | 103483 | 102674 | + | head maturation protease | head maturation protease [Escherichia phage T4] | 99.5 | 1.00E-151 | NP_049785.1 |
| 172 | 105067 | 103502 | + | head scaffolding protein | head scaffolding protein [Escherichia phage vB_EcoM_G50] | 100 | 0 | YP_010070001.1 |
| 173 | 105117 | 105383 | - | major head protein | major head protein [Escherichia phage vB_EcoM_SA20RB] | 99.2 | 0 | UIU28117.1 |
| 175 | 106799 | 107803 | - | head vertex subunit precursor | head vertex subunit precursor [Escherichia phage RB32] | 99.5 | 0 | YP_803116.1 |

| | | | | | | | | |
|---|---|---|---|---|---|---|---|---|
| 179 | 109243 | 109923 | - | capsid and scaffold protein | capsid and scaffold protein [Escherichia phage EP01] | 94 | 0 | UIU46927.1 |
| 180 | 111485 | 109974 | + | minor head protein inhibitor of protease | minor head protein inhibitor of protease [Salmonella phage SG1] | 100 | 5.00E-162 | YP_010075074.1 |
| 186 | 113095 | 113721 | - | baseplate wedge subunit | baseplate wedge subunit [Shigella phage ESh36] | 99.2 | 1.00E-89 | URY16176.1 |
| 187 | 114521 | 113772 | + | baseplate hub | baseplate hub [Escherichia phage vB_EcoM_KAW1E185] | 100 | 1.00E-149 | YP_010071392.1 |
| 188 | 115696 | 114521 | + | baseplate hub | baseplate hub assembly protein [Shigella phage Sf24] | 100 | 0 | YP_009619120.1 |
| 189 | 116174 | 115716 | + | baseplate hub | baseplate hub [Phage NBEco003] | 99.7 | 0 | YP_010106060.1 |
| 190 | 117943 | 116171 | + | baseplate distal hub subunit | baseplate distal hub subunit [Escherichia phage N2] | 100 | 9.00E-107 | UTQ79510.1 |
| 191 | 119046 | 117952 | + | baseplate tail tube cap | tail-tube assembly protein [Escherichia phage W143] | 99.5 | 0 | QWV60538.1 |
| 192 | 120011 | 119046 | + | tail tube | tail tube [Enterobacteria phage vB_EcoM_IME340] | 99.7 | 0 | YP_010066461.1 |
| 208 | 130362 | 130697 | - | head morphogenesis | head morphogenesis [Serratia phage PhiZZ30] | 98.2 | 2.00E-72 | YP_010075749.1 |
| 210 | 131063 | 131299 | - | tail fibers protein | tail fibers protein [Escherichia phage UTI-E4] | 98.7 | 4.00E-49 | UMM76439.1 |
| 238 | 151509 | 147640 | + | long tail fiber proximal subunit | long tail fiber proximal subunit [Escherichia phage EP01] | 97 | 0 | UIU46991.1 |
| 239 | 152633 | 151518 | + | long tail fiber protein proximal connector | long tail fiber protein proximal connector [Citrobacter phage vB_CroM_CrRp10] | 99.5 | 0 | YP_010065303.1 |
| 240 | 153358 | 152696 | + | putative tail connector protein | putative tail connector protein [Hafnia phage vB_HpaM_IsaacDaniel] | 99.2 | 0 | QXN69512.1 |

| | | | | | | | | |
|---|---|---|---|---|---|---|---|---|
| 241 | 157278 | 153367 | + | long tail fiber protein distal subunit | long tail fiber protein distal subunit [Escherichia phage REP4] | 83 | 0 | WBY53099.1 |
| 242 | 158100 | 157309 | + | receptor recognizing protein | receptor recognizing protein [Shigella phage TB004] | 99.6 | 1.00E-179 | QTZ59846.1 |
| 260 | 164728 | 164826 | - | outer membrane protein | outer membrane protein [Escherichia phage wV7] | 100 | 1.00E-12 | YP_007005010.1 |
| **Lysis modules** | | | | | | | | |
| 36 | 18237 | 18530 | - | spackle periplasmic | spackle periplasmic [Escherichia phage teqhad] | 99 | 7.00E-65 | YP_010074241.1 |
| 107 | 57770 | 58063 | - | lysis inhibition | lysis inhibition [Enterobacteria phage RB51] | 99 | 4.00E-65 | YP_002854065.1 |
| 207 | 129966 | 130214 | - | lysis inhibition accessory protein | lysis inhibition; accessory protein [Escherichia phage teqdroes] | 98.9 | 7.00E-51 | YP_010074086.1 |
| 243 | 158787 | 158131 | + | holin | putative holin [Hafnia phage vB_HpaM_IsaacDaniel] | 99.5 | 3.00E-160 | QXN69516.1 |
